# Supplementary material for: Evaluating the prevalence and risk factors for macrolide resistance in Mycoplasma genitalium using a newly developed qPCR assay
Source: PLoS One. 2020 Oct 20;15(10):e0240836. doi: 10.1371/journal.pone.0240836 (PMC7575077; doi:10.1371/journal.pone.0240836)
Supplement: S1 Table — (DOCX) [file pone.0240836.s001.docx]

**S1 Table. *Mycoplasma Genitalium* (MG) detection by different molecular techniques and according to anatomical location.**

| **Anatomical location** |  | **MgPa positive** | **MgPa negative** | **Total^1^** |
| --- | --- | --- | --- | --- |
| **Vagina** | **MG-MRAM positive** | 84 (61.8%) | 7 (5.1%) | 91 (66.9%) |
|  | **MG-MRAM negative** | 15 (11.0%) | 30 (22.1%) | 45 (33.1%) |
|  | **Total** | 99 (72.8%) | 37 (27.2%) | 136 (100%) |
| **Urine** | **MG-MRAM positive** | 64 (53.3%) | 8 (6.7%) | 72 (60.0%) |
|  | **MG-MRAM negative** | 8 (6.7%) | 40 (33.3%) | 48 (40.0%) |
|  | **Total** | 72 (60.0%) | 48 (40.0%) | 120 (100%) |
| **Anus** | **MG-MRAM positive** | 118 (49.8%) | 28 (11.8%) | 146 (61.6%) |
|  | **MG-MRAM negative** | 16 (6.8%) | 75 (31.6%) | 91 (38.4%) |
|  | **Total** | 134 (56.5%) | 103 (43.5%) | 237 (100%) |
| **All samples** | **MG-MRAM positive** | 266 (54.0%) | 43 (8.7%) | 309 (62.7%) |
|  | **MG-MRAM negative** | 39 (7.9%) | 145 (29.4%) | 184 (37.3%) |
|  | **Total^1^** | 305 (61.9%) | 188 (38.1%) | 493 (100%) |

^1^ Total samples represent samples that were previously positive tested for MG with the TMA assay.
There was a substantial concordance between MgPa qPCR and MG-MRAM qPCR with a Cohen’s kappa for agreement of 0.646.
