# Supplementary material for: Evaluating the prevalence and risk factors for macrolide resistance in Mycoplasma genitalium using a newly developed qPCR assay
Source: PLoS One. 2020 Oct 20;15(10):e0240836. doi: 10.1371/journal.pone.0240836 (PMC7575077; doi:10.1371/journal.pone.0240836)
Supplement: S2 Table — (DOCX) [file pone.0240836.s002.docx]

**S2 Table. Comparison of MG-MRAM qPCR and sequencing analysis to detect MRAM in MG.**

|  |  | **Sequencing** | | |  |
| --- | --- | --- | --- | --- | --- |
|  |  | **MRAM** | **WT** | **Total** | |
| **MG-MRAM qPCR** | **MRAM** | 68 | 2 | 70 | |
|  | **WT** | 1 | 32 | 33 | |
|  | **Total** | 69 | 34 | 103 | |

From 103 of 126 samples that were typed with the MG-MRAM qPCR sequences could also be determined. Sensitivity = 98.6% (68/69; 95%CI 91.1%-99.9%) and specificity = 94.1% (32/34; 95%CI 78.9%-99.0%).
