# Supplementary material for: Evaluating the prevalence and risk factors for macrolide resistance in Mycoplasma genitalium using a newly developed qPCR assay
Source: PLoS One. 2020 Oct 20;15(10):e0240836. doi: 10.1371/journal.pone.0240836 (PMC7575077; doi:10.1371/journal.pone.0240836)
Supplement: S3 Table — (DOCX) [file pone.0240836.s003.docx]

**S3 Table. Prevalence of mutant MG (MG-MRAM) as percentage of typeable samples according to different client characteristics separately for men and women.**

|  |  | Women (typeable) | Mutants (% of typeable) | p-value^2^ | Men  (typeable) | Mutants (% of typeable) | p-value^2^ |
| --- | --- | --- | --- | --- | --- | --- | --- |
| Overall^1^ |  | 113 | 64 (56.6%) |  | 178 | 129 (72.5%) |  |
|  |  |  |  |  |  |  |  |
|  |  |  |  |  |  |  |  |
| Age in years | <25 | 82 | 45 (54.9%) | 0.481 | 33 | 24 (72.7%) | 0.517 |
|  | 25-34 | 24 | 16 (66.7%) |  | 69 | 47 (68.1%) |  |
|  | 35-44 | 6 | 3 (50.0%) |  | 38 | 31 (81.6%) |  |
|  | >=45 | 1 | 0 |  | 38 | 27 (71.1%) |  |
|  |  |  |  |  |  |  |  |
| Ethnicity | Dutch | 68 | 40 (58.8%) | 0.440 | 91 | 63 (69.2%) | 0.560 |
|  | Other European | 13 | 8 (61.5%) |  | 24 | 21 (87.5%) |  |
|  | African | 4 | 1 (25.0%) |  | 16 | 11 (68.8%) |  |
|  | Mid/South American | 15 | 6 (40.0%) |  | 31 | 23 (74.2%) |  |
|  | Asian | 11 | 7 (63.6%) |  | 11 | 7 (63.6%) |  |
|  | Other | 2 | 2 (100%) |  | 5 | 4 (80.0%) |  |
|  |  |  |  |  |  |  |  |
| Educational level | Low | 12 | 4 (33.3%) | 0.145 | 18 | 12 (66.7%) | 0.097 |
|  | Mid | 36 | 20 (55.6%) |  | 52 | 32 (61.5%) |  |
|  | High | 58 | 37 (63.8%) |  | 99 | 77 (77.8%) |  |
|  |  |  |  |  |  |  |  |
| HIV | Negative | 113 | 64 (56.6%) | NA | 148 | 104 (70.3%) | 0.256 |
|  | Positive | 0 | 0 |  | 29 | 24 (82.8%) |  |
|  |  |  |  |  |  |  |  |
| Chlamydia | Negative | 98 | 56 (57.1%) | 0.782 | 155 | 112 (72.3%) | 0.868 |
|  | Positive | 15 | 8 (53.3%) |  | 23 | 17 (73.9%) |  |
|  |  |  |  |  |  |  |  |
| Gonorrhea | Negative | 111 | 62 (55.9%) | 0.504 | 154 | 110 (71.4%) | 0.430 |
|  | Positive | 2 | 2 (100%) |  | 24 | 19 (79.2%) |  |
|  |  |  |  |  |  |  |  |
| Sexual preference | MSM | NA | NA | NA | 134 | 99 (73.9%) | 0.463 |
|  | Hetero | NA | NA | NA | 44 | 30 (68.2%) |  |
|  |  |  |  |  |  |  |  |
| Reported having performed sex | No | 101 | 57 (56.4%) | 0.900 | 170 | 123 (72.4%) | 1.000 |
| work in preceding 6 months | Yes | 12 | 7 (58.3%) |  | 6 | 5 (83.3%) |  |
|  |  |  |  |  |  |  |  |
| Azithromycin in previous 3 | No | 111 | 62 (55.9%) | 0.504 | 174 | 126 (72.4%) | 1.000 |
| months | Yes | 2 | 2 (100%) |  | 4 | 3 (75%) |  |
|  |  |  |  |  |  |  |  |
| No. of sexual partners in | 0-2 | 46 | 27 (58.7%) | 0.866 | 30 | 17 (56.7%) | 0.037 |
| previous 6 months | 3-10 | 56 | 31 (55.4%) |  | 87 | 62 (71.3%) |  |
|  | >10 | 10 | 5 (50.0%) |  | 61 | 50 (82.0%) |  |
|  |  |  |  |  |  |  |  |
| Any symptom | No | 88 | 49 (55.7%) | 0.701 | 126 | 89 (70.6%) | 0.393 |
|  | Yes | 25 | 15 (60.0%) |  | 52 | 40 (76.9%) |  |
|  |  |  |  |  |  |  |  |
| Urogenital discharge | No | 96 | 54 (56.2%) | 0.844 | 148 | 107 (72.3%) | 0.908 |
|  | Yes | 17 | 10 (58.8%) |  | 30 | 22 (73.3%) |  |
|  |  |  |  |  |  |  |  |
| Dysuria | No | 103 | 57 (55.3%) | 0.372 | 149 | 110 (73.8%) | 0.359 |
|  | Yes | 10 | 7 (70.0%) |  | 29 | 19 (65.5%) |  |
|  |  |  |  |  |  |  |  |
| Ulcers | No | 112 | 64 (56.6%) | 0.434 | 175 | 126 (72.0%) | 0.562 |
|  | Yes | 1 | 0 (0%) |  | 3 | 3 (100.0%) |  |
|  |  |  |  |  |  |  |  |
| Blood loss | No | 106 | 60 (56.6%) | 1.000 | 178 | 129 (72.5%) | N.A. |
|  | Yes | 7 | 4 (57.1%) |  | N.A. | N.A. |  |
|  |  |  |  |  |  |  |  |
| Pain | No | 110 | 62 (56.4%) | 1.000 | 178 | 129 (72.5%) | N.A. |
|  | Yes | 3 | 2 (66.7%) |  | 0 | N.A. |  |

^1^A client was considered to be infected with mutant MG (MG-MRAM) if in at least one of the samples a mutation was detected. ^2^Overall p-value determined with Chi-square test or Fischer exact test when expected value in >=1 cell is <5. NA: not applicable, no p-value could be calculated, since all HIV-positive clients were male and sexual preference was not asked for women.
